# Symbiosis between the Javan rhinoceros and slender‐billed crow: A novel inferred cleaning mutualism

**DOI:** 10.1002/ece3.70224

**Published:** 2024-09-01

**Authors:** Montana M. Stone, Herry Afriandi, Firmanto Noviar Suwanda, Ardi Andono, Rois Mahmud, Oktavia K. Khairani, Anne B. Clark, Michael Webster, Kevin McGowan, Robin W. Radcliffe

**Affiliations:** ^1^ College of Arts & Sciences, Cornell University Ithaca New York USA; ^2^ Department of Ecology and Evolutionary Biology Brown University Providence Rhode Island USA; ^3^ Asian Rhinoceros Special Species Group International Union for Conservation of Nature Gland Switzerland; ^4^ Ujung Kulon National Park Ujungjaya Banten Indonesia; ^5^ Alliance for Integrated Forest Conservation Bogor Jawa Barat Indonesia; ^6^ State University of New York Binghamton New York USA; ^7^ Cornell Lab of Ornithology, Cornell University Ithaca New York USA; ^8^ Cornell Conservation Medicine Program College of Veterinary Medicine, Cornell University Ithaca New York USA

**Keywords:** behavioral convergent evolution, cleaning mutualism, novel symbiosis, rhinoceros conservation

## Abstract

Over the past century, the Javan rhinoceroses' (*Rhinoceros sondaicus*) secluded nature and low population size have led to a gap in knowledge of their ecology. With fewer than 80 individuals surviving in a single population in West Java, Indonesia, the Javan rhinoceros is one of the most critically endangered mammals in the world. As part of a pilot bioacoustics study of the Javan rhinoceros in 2019, we systematically reviewed camera trap footage from the core Javan rhinoceros range in Ujung Kulon National Park (UKNP). In doing so, we discovered a previously unknown interaction between the Javan rhinoceros and the slender‐billed crow (*Corvus enca*), in which the crow finds and eats ectoparasites from the rhinoceros (Figure 1). We describe this interaction and suggest that it may represent a cleaning mutualism with benefits for both the crow and the rhinoceros.

## Graphical abstract

As part of a pilot bioacoustics study of the Javan rhinoceros in 2019, we systematically reviewed camera trap footage from the core Javan rhinoceros range in Ujung Kulon National Park (UKNP) and discovered a previously unknown interaction between the Javan rhinoceros and the slender‐billed crow (*Corvus enca*), in which the crow finds and eats ectoparasites from the rhinoceros (Figure 1). We describe this interaction, investigate it through an ecological and evolutionary lens, and suggest that it may represent a cleaning mutualism with conservation benefits for both the crow and the rhinoceros.

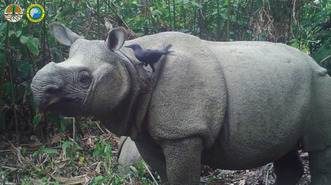

Javan rhinoceros (*Rhinoceros sondaicus*) are one of the most endangered animals in the world yet, little is known about their basic biology because of their low population size and secluded nature (Margaryan et al., 2020; Putra et al., 2020). Camera traps were initially placed in UKNP in 2009 to monitor rhinoceros populations, and video data is systematically cataloged and stored by the park. Camera trap footage recorded from April to September of 2018 at 21 different sites within UKNP was reviewed for this report. During the study period, ninety‐nine observations of Javan rhinoceroses were recorded and made available to researchers. Of those ninety‐nine recordings, the direct interaction between the Javan rhinoceros and slender‐billed crow (*Corvus enca*) was only recorded once, for over 30 min via camera trap on September 23, 2018 in UKNP on the southern coastal border at a known rhinoceros trail (UKNP Archive). Physical interaction between these two species has not been previously described in the literature. The crow species was identified by bill shape, location, and behavior.

During the recorded interaction, two slender‐billed crows land on, and scavenge ectoparasites from, an adult female and a juvenile male rhinoceros. The slender‐billed crows initially fly from the higher canopy of the rainforest onto the rhinoceroses' backs and later approach the rhinoceroses from the ground. The crows focus on three ectoparasite‐rich regions of the rhinoceros: the skin folds of the back and neck (Figure 1a), the ears (Figure 1b), and the inguinal region. Between probes into the skin folds of the rhinoceros, the crows visibly swallow, indicating that they are successfully scavenging food (presumably ectoparasites) from the rhinoceros. The recorded interaction between the crows and rhinoceroses lasts 30 minutes with 386 seconds of visible physical contact between the crows and the rhinoceroses. Since the crows were not individually identified from the video segments, pooled observations for the crows are described. Timed behavioral observation of both crows revealed 15 s with a crow's head inside the rhinoceroses' ear, 36 s moving around the rhinoceroses' head, 283 s investigating and probing its beak into the skin folds of the rhinoceros's neck, and 52 s traversing the inguinal region. The crows land on and interact with both the calf and mother rhinoceros, spending 78 s or 20.2% of time on the calf and 308 s, or 79.8% of time on the mother.

**FIGURE 1:**
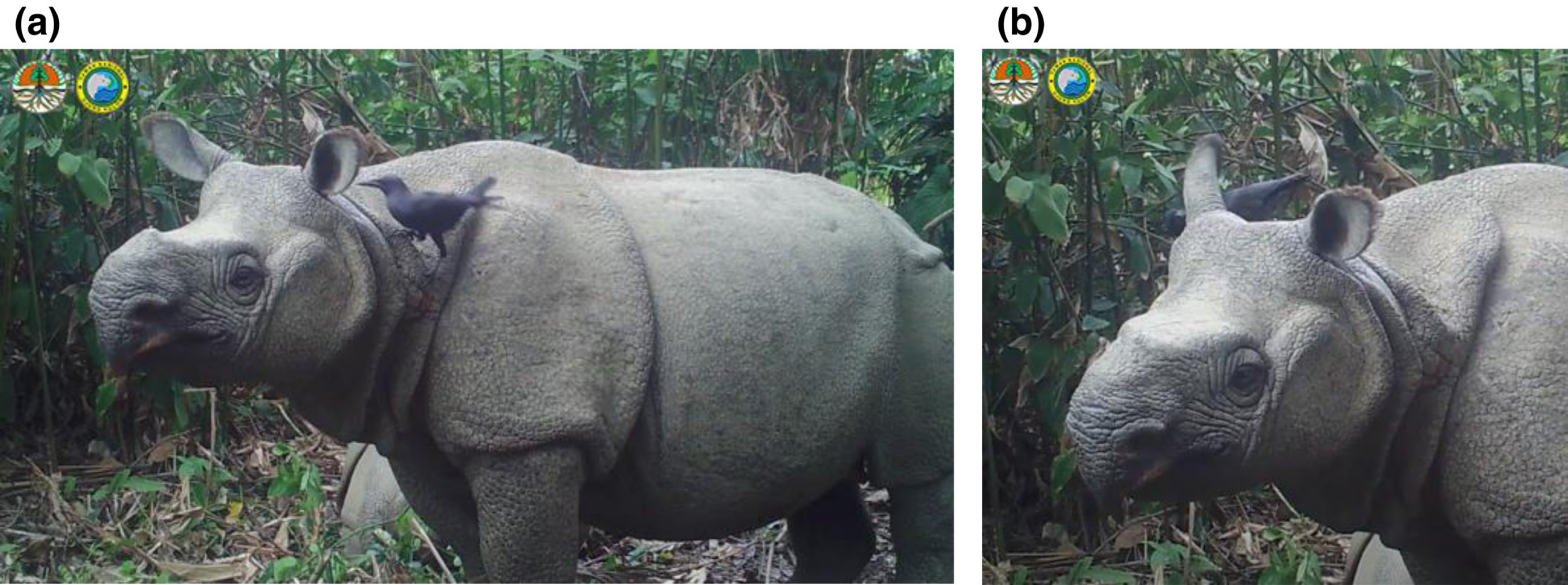
(a) On September 23, 2018 in Ujung Kulon National Park, a slender‐billed crow (*Corvus enca*) examines the ear of a female Javan rhinoceros (*Rhinoceros sondaicus*) in search of ectoparasites (presumably ticks and leeches) in a case of apparent symbiotic mutualism. (b) A female Javan rhinoceros (*Rhinoceros sondaicus*) pivots her ear to solicit parasite removal by a slender‐billed crow (*Corvus enca*).

In two separate instances during the interaction, the adult female rhinoceros pivots her ear toward the crow, which then sticks its head and bill into the ear followed by swallowing motions (Figure 1). The rhinoceros' behavior does not change when the crow's head enters the rhinoceros' ear. The level of coordination in this interaction suggests a familiarity of these two species in doing this behavior, a high level of reward for both individuals with this kind of symbiosis, or both.

From our experience with African and Sumatran rhinoceros, the rhinoceros' skin folds, tail, and ears are hosts to many ectoparasites, especially ticks (and sometimes leeches) (Figure 2) (Penzhorn & Nicolaas, 1994); both species are known to host a range of ectoparasite‐borne diseases including *Babesia*, *Theileria*, and *Anaplasma* (Nijhof et al., 2003, 2021). Of those, *Anaplasma phagocytophilum* is a zoonosis of high morbidity in animals if not diagnosed and treated. Periodic die‐offs of Javan rhinoceros since the 1980s have drastically reduced the species' population (for example, five individuals of the remaining 60 Javan rhinoceros died of anthrax or an anthrax like disease in 1981 (Schenkel & Schenkel, 1982)) and are thought to be manifestations of larger infectious disease outbreaks in the region involving vector‐borne pathogens (Khairani et al., 2018; Radcliffe & Khairani, 2019). In many cases, the specific cause of Javan rhinoceros die‐offs could not be determined with certainty.

**FIGURE 2:**
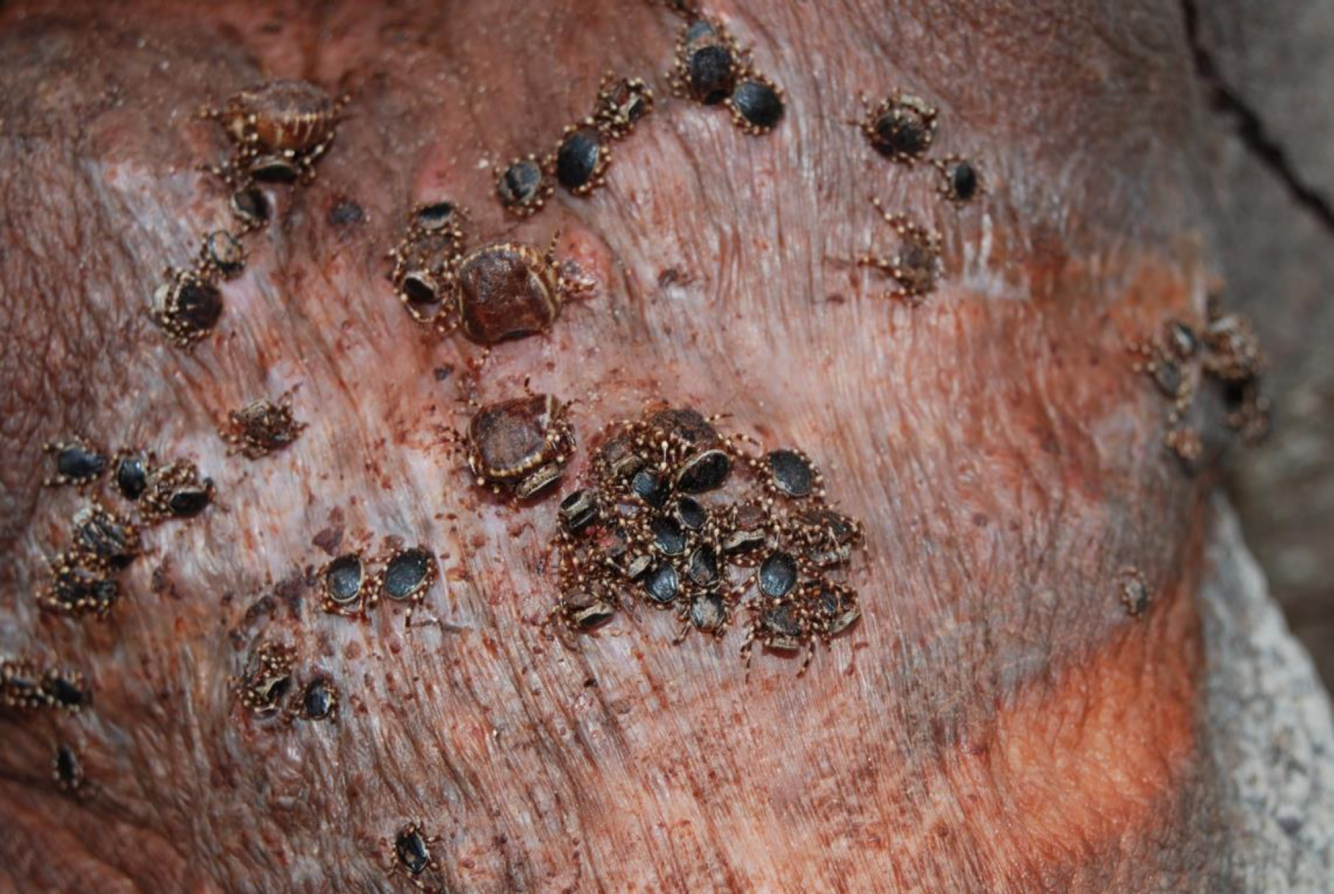
The inguinal region of a black rhinoceros (*Diceros bicornis*) displaying a variety of ticks. Similar folds of the Javan rhinoceros (*Rhinoceros sondaicus*) almost certainly harbor similar ectoparasite burdens since ticks are abundant on sympatric Sumatran rhinoceroses (*Dicerorhinus sumatrensis*) that are managed in sanctuaries in Indonesia.

Simultaneous with our discovery of a potential mutualism between the crow and Javan rhinoceros, we learned from local village leaders that a similar cleaning interaction between the endemic Javan Mynah (*Acridotheres javanicus*) and domesticated water buffalo (*Bubalus bubalis*) was regularly observed before the mynah was extirpated from UKNP. The presence of these symbioses in UKNP suggests that at some point in rhinoceros evolutionary history, the threat of ticks and tick‐borne disease may have acted as a driver for rhinoceroses to tolerate a large bird landing on their bodies for parasite removal, an apparent symbiosis in the Javan rhinoceros that has never been described before. The slender‐billed crow cleaning behavior may offer health benefits for these rare forest rhinoceroses by reducing ticks and tick‐borne disease burdens. However, since the crows are also reported by local residents to feed on and around domestic water buffalo that surround the park and birds are known to transport ticks between hosts (Hasle, 2013), disease sharing between domestic and wild ungulates because of the crow association is also possible.

This inferred Javan rhinoceros—slender‐billed crow cleaning symbiosis is not unlike the relationship between the African black rhinoceros (*Diceros bicornis*) and white rhinoceros (*Ceratotherium simum*) and the red‐billed oxpecker (*Buphagus erythrorhynchus*) and the yellow‐billed oxpecker (*Buphagus africanus*), where the oxpeckers eat ectoparasites from the skin folds of the rhinoceros (Bishop & Bishop, 2014). The oxpeckers living close to black and white rhinoceroses may also offer an “early warning signal” for surveillance against predators in the wild (Plotz & Linklater, 2020). The mutualistic nature of the oxpecker‐African rhinoceros symbiosis has been debated; some argue that the oxpeckers may create new wounds and eat the tissue of the ungulates from which they scavenge ectoparasites, which would be a negative outcome for the rhinoceros (Bishop & Bishop, 2014; McElligott et al., 2004). These interactions may border on parasitism with wound‐feeding and are context‐dependent, often changing with geographic location or habitat (captivity vs. wild), presence of hemorrhagic firial lesions (Plotz, 2014), or density of ectoparasites (Cheney & Côté, 2005; Plantan, 2009; Plantan et al., 2013). In some cases, cleaning mutualisms transfer diseases between host species (Keet et al., 1997). Likewise, the inferred cleaning mutualism between the Javan rhinoceros and slender‐billed crow may vary in magnitude and direction depending on the biological context and may have significant effects on parasite ecology and disease dynamics (Hopkins et al., 2017).

Ectoparasite cleaning interactions between birds and ungulates are increasingly documented (Sazima et al., 2012; Sazima & Sazima, 2010), and more specifically, other Corvid species are partners in several ectoparasite cleaning interactions. American crows (*Corvus brachyrhynchos*) have been known to land on and clean ectoparasites from wild pigs (*Sus scrofa*) and cattle (*Bos* spp.) in Florida (Kilham, 1982); Carrion crows (*Corvus corone*) land on and clean wild boar (*Sus scrofa*) in Italy (Massei & Genov, 1995); and Torresian crows (*Corvus orru*) land on and clean introduced banteng (*Bos javanicus*) in Australia (Bradshaw & White, 2006). A common thread for these crow and ungulate interactions is that they all exhibit a degree of behavioral facilitation from the cleaned animals. These relationships suggest a convergent evolution of behavior, in which there is a mutualism between scavenging birds and ungulates. The birds benefit from nutrient‐rich foods and perhaps protection from larger predators, while the ungulates benefit from protection from tick‐borne diseases and an early alarm system to avoid predation. Although there is little research into the biogeography of the slender‐billed crow (Oortwijn, 1987), we have no evidence to suggest that the crows and rhinoceroses have not coexisted for at least some part of their evolutionary history.

Our limited observations of slender‐billed crow and Javan rhinoceros behavior suggest that the relationship may be more mutualistic than parasitic based on the following evidence: (1) Javan rhinoceroses lack hemorrhaging external wounds from filarial parasites like their African counterparts; (2) Javan rhinoceroses show minimal resistance behaviors (occasional head shake, but nothing more); and (3) Javan rhinoceroses show apparent solicitation behaviors (pivoting ear) not unlike that described for impala and zebra with their oxpecker symbionts (Breitwisch, 1992; Mooring & Mundy, 1996). While we did not observe any wound‐feeding from the slender‐billed crow on the Javan rhinoceros, we cannot rule out that the crows may be targeting the skin folds because of lesions or creating lesions opportunistically.

Knowledge of Javan rhinoceros ecology is extremely limited, and a single interaction cannot be used to define this behavior as a cleaning mutualism. Yet, this inferred cleaning symbiosis may have important conservation implications for the Javan rhinoceros because a proposed second population of individuals is awaiting translocation by the Indonesian government to a second protected area (Wilson, 2021). The creation of a second population of Javan rhinoceroses outside of UKNP may be one of the most critical factors for the survival of the species as a whole. Furthermore, ticks and tick‐borne disease are a recognized concern for both Asian and African rhinoceroses, and disease has resulted in failure of rhinoceros reintroduction and translocation programs (Fyumagwa et al., 2004; Nijhof et al., 2003, 2021). The slender‐billed crow may represent an important part of a natural ecological cycle for controlling rhinoceros tick burdens in the rainforests of Indonesia and, like their African oxpecker counterparts (Bishop & Bishop, 2014; Plantan et al., 2013), may also represent a warning signal to the rhinoceroses for predators or poachers. New understanding of these interactions could guide future translocation efforts to ensure that both the rhinoceroses and the crows are a part of any second habitat for the Javan rhinoceros. Therefore, future research should expand our understanding of Asian rhinoceros parasites, this interaction, its role in a natural cycle of parasite‐borne disease prevention, and as a warning signal to the rhinoceros. With international communities and the IUCN pushing for a second habitat for the Javan rhinoceros, potential symbiotic relationships like these should be considered as part of any species relocation effort.

The next ten years will decide the future of the critically endangered Javan rhinoceros, a fate that is intimately connected to its ecology. This heretofore unknown cleaning symbiosis demonstrates how preserving one of the most critically endangered species in the world requires a comprehensive exploration of its ecology, specifically its ecological community relationships, in order to ensure that the most effective conservation strategies are identified.

## AUTHOR CONTRIBUTIONS

**Montana M. Stone:** Conceptualization (lead); project administration (supporting); writing – original draft (equal). **Herry Afriandi:** Data curation (equal); writing – review and editing (equal). **Firmanto Noviar Suwanda:** Data curation (equal); writing – review and editing (supporting). **Ardi Andono:** Data curation (lead); writing – review and editing (supporting). **Rois Mahmud:** Conceptualization (equal); project administration (equal); writing – review and editing (equal). **Oktavia K. Khairani:** Project administration (equal); writing – review and editing (equal). **Anne B. Clark:** Conceptualization (equal); investigation (equal); writing – original draft (equal); writing – review and editing (equal). **Michael Webster:** Writing – review and editing (equal). **Kevin McGowan:** Investigation (equal); writing – review and editing (equal). **Robin W. Radcliffe:** Conceptualization (equal); writing – original draft (equal); writing – review and editing (lead).

## CONFLICT OF INTEREST STATEMENT

The authors declare no conflicts of interest.

## Data Availability

Data are not yet provided due to the active risk of poaching in the area and metadata stored on the raw files. Upon acceptance data will be archived in the wildlife archive at https://www.macaulaylibrary.org and https://tnujungkulon.menlhk.go.id.

## References

[ece370224-bib-0001] Bishop, A. L. , & Bishop, R. P. (2014). Resistance of wild African ungulates to foraging by red‐billed oxpeckers (*Buphagus erythrorhynchus*): Evidence that this behaviour modulates a potentially parasitic interaction. African Journal of Ecology, 52(1), 103–110.

[ece370224-bib-0002] Bradshaw, C. , & White, W. W. (2006). Rapid development of cleaning behaviour by Torresian crows *Corvus orru* on non‐native banteng *Bos javanicus* in northern Australia. Journal of Avian Biology, 37(4), 409–411.

[ece370224-bib-0003] Breitwisch, R. (1992). Tickling for ticks. Natural History, 101(3), 56–63.

[ece370224-bib-0004] Cheney, K. L. , & Côté, I. M. (2005). Mutualism or parasitism? The variable outcome of cleaning symbioses. Biology Letters, 1(2), 162–165. 17148155 10.1098/rsbl.2004.0288 PMC1626222

[ece370224-bib-0005] Fyumagwa, R. D. , Mkumbo, H. S. , & Morkel, P. B. (2004). Remote treatment of black rhinoceroses against babesiosis in Ngorongoro Crater, Tanzania. Pachyderm, 37, 80–83.

[ece370224-bib-0006] Hasle, G. (2013). Transport of ixodid ticks and tick‐borne pathogens by migratory birds. Frontiers in Cellular and Infection Microbiology, 3(48), 1–6. 24058903 10.3389/fcimb.2013.00048 PMC3767891

[ece370224-bib-0007] Hopkins, S. R. , Wojdak, J. M. , & Belden, L. K. (2017). Defensive symbionts mediate host‐parasite interactions at multiple scales. Trends in Parasitology, 33(1), 53–64. 27810464 10.1016/j.pt.2016.10.003

[ece370224-bib-0008] Keet, D. F. , Kriek, J. , Zakrisson, G. , Meltzer, D. G. A. , & Boomker, J. D. F. (1997). Parafilariosis in African buffaloes (*Syncerus caffer*). 9467178

[ece370224-bib-0009] Khairani, K. O. , Daryl Nydam, M. , Felippe, J. , McDonough, P. , Barry, J. , Mahmud, R. , Haryono, M. , & Radcliffe, R. W. (2018). Surveillance for hemorrhagic septicemia in buffalo (*Bubalus bubalis*) as an aid to range expansion of the Javan rhinoceros (*Rhinoceros sondaicus*) in Ujung Kulon National Park, Indonesia. Journal of Wildlife Diseases, 54(1), 14–25. 28820298 10.7589/2015-07-183

[ece370224-bib-0010] Kilham, L. (1982). Cleaning/feeding symbioses of common crows with cattle and feral hogs. Journal of Field Ornithology, 53(3), 275–276.

[ece370224-bib-0011] Margaryan, A. , Sinding, M.‐H. S. , Liu, S. , Vieira, F. G. , Chan, Y. L. , Nathan, S. K. S. S. , Moodley, Y. , Bruford, M. W. , Thomas, M. , & Gilbert, P. (2020). Recent mitochondrial lineage extinction in the critically endangered Javan rhinoceros. Zoological Journal of the Linnean Society, 190(1), 372–383.

[ece370224-bib-0012] Massei, G. , & Genov, P. (1995). Observations of black‐billed magpie (*Pica pica*) and carrion crow (*Corvus corone comix*) grooming wild boar (*Sus scrofa*). Journal of Zoology, 236(2), 338–341.

[ece370224-bib-0013] McElligott, A. G. , Maggini, I. , Hunziker, L. , & König, B. (2004). Interactions between red‐billed oxpeckers and black rhinoceroses in captivity. Zoo Biology, 23(4), 347–354.

[ece370224-bib-0014] Mooring, M. S. , & Mundy, P. J. (1996). Interactions between impala and oxpeckers at Matobo National Park, Zimbabwe. African Journal of Ecology, 34, 54–65.

[ece370224-bib-0015] Nijhof, A. M. , Penzhorn, B. L. , Lynen, G. , Mollel, J. O. , Morkel, P. , Bekker, C. P. J. , & Jongejan, F. (2003). *Babesia bicornis* sp. nov. and *Theileria bicornis* sp. nov.: Tick‐borne parasites associated with mortality in the black rhinoceros (*Diceros bicornis*). Journal of Clinical Microbiology, 41(5), 2249–2254. 12734294 10.1128/JCM.41.5.2249-2254.2003 PMC154668

[ece370224-bib-0016] Nijhof, A. M. , Radcliffe Robin, W. , Candra, D. , Agil, M. , Ramono, W. , Adi, M. , van Strien, N. , Foose, T. , Florin‐Christensen, M. , & Schnittger, L. (2021). A novel *Theileria* species infecting the Sumatran Rhinoceros (*Dicerorhinus sumatrensis*): Evidence for piroplasmid‐host cospeciation. 14th International Symposium on Ticks and Tick‐borne Diseases. Zurich, Germany March 24–26, 2021 (Digital programme).

[ece370224-bib-0017] Oortwijn, R. G. M. (1987). Geographic variation and subspecies of *Corvus enca* (Horsfield, 1821). Zoologische Mededelingen, 61(3), 31–51.

[ece370224-bib-0018] Penzhorn, B. L. , & Nicolaas, P. J. K. (1994). Proceedings of a Symposium on Rhinoceroses as Game Ranch Animals, Onderstepoort, Republic of South Africa, 9–10 September 1994. In *Symposium on Rhinoceroses as Game Ranch Animals* (*1994: Onderstepoort, Pretoria, South Africa*). Wildlife Group, South African Veterinary Association in collaboration with the Wildlife Research Programme, Faculty of Veterinary Science, University of Pretoria.

[ece370224-bib-0019] Plantan, T. B. (2009). Feeding behavior of wild and captive oxpeckers (*Buphagus* spp.): A case of conditional mutualism. Diss.

[ece370224-bib-0020] Plantan, T. , Howitt, M. , Kotzé, A. , & Gaines, M. (2013). Feeding preferences of the red‐billed oxpecker, *Buphagus erythrorhynchus*: A parasitic mutualist? African Journal of Ecology, 51(2), 325–336.

[ece370224-bib-0022] Plotz, R. D. (2014). The interspecific relationships of black rhinoceros (*Diceros bicornis*) in Hluhluwe‐iMfolozi Park. Diss. Open Access Te Herenga Waka‐Victoria University of Wellington.

[ece370224-bib-0021] Plotz, R. D. , & Linklater, W. L. (2020). Oxpeckers help rhinoceroses evade humans. Current Biology, 30(10), 1965–1969. 32275876 10.1016/j.cub.2020.03.015

[ece370224-bib-0023] Putra, W. P. , Bayu, M. S. , & Firdaus, A. Y. (2020). Population structure analysis of Javan rhinoceros at Ujung Kulon National Park, West Java. Buletin Plasma Nutfah, 26(2), 103–108.

[ece370224-bib-0024] Radcliffe, R. W. , & Khairani, K. O. (2019). Health of the forest rhinoceros of southeast Asia: Sumatran and Javan rhinoceros. Fowler's Zoo and Wild Animal Medicine Current Therapy, 9, 707–715.

[ece370224-bib-0025] Sazima, C. , Jordano, P. , Guimarães, P. R., Jr. , Dos Reis, S. F. , & Sazima, I. (2012). Cleaning associations between birds and herbivorous mammals in Brazil: Structure and complexity. The Auk, 129(1), 36–43.

[ece370224-bib-0026] Sazima, I. , & Sazima, C. (2010). Cleaner birds: An overview for the Neotropics. Biota Neotropica, 10, 195–203.

[ece370224-bib-0027] Schenkel, R. , & Schenkel, L. (1982). Mystery of dead Javan rhinoceroses. WWF/IUCN Service, 2(3), 265.

[ece370224-bib-0028] Wilson, S. G. (2021). Factors shaping the conservation of the critically endangered Javan Rhinoceros *Rhinoceros sondaicus* .

